# Are We to Have Cholera in the United States in 1885?

**Published:** 1885-03

**Authors:** P. P. Wells


### HOMŒOPATHIC PHYSICIAN,

A MONTHLY JOURNAL OF MEDICAL SCIENCE.

"If our school ever gives up the strict inductive method of Hahnemann, we are lost, and deserve only to be mentioned as a caricature in the history of medicine."—CONSTANTINE HERING.

Vol. V. MARCH, 1885. No. 3.

### ARE WE TO HAVE CHOLERA IN THE UNITED STATES IN 1885?

If this be answered in the affirmative (this is at least possible), then is it too early to ask another question: What, before this future possibility, is the present duty of doctors and men? The answer to this last, if we come to details of duty, will be determined by the views of the respondent as to the nature of the cause, of the disease itself, and of the agents employed for its cure. In general, it may be said, and all will agree to it, the duty is to prevent its approach in the first place. This is to be met by proper quarantine regulations (the duty of the Government), by proper sanitation (the duty of the municipal authorities), and by prophylaxis (to be discovered and administered by the doctors) when the disease is really present.

First, as to the nature of the cause? It is now more than half a century since cholera intruded itself on the attention of the physicians of the civilized world, and gave them opportunity to study its own nature and that of its cause. Repeated epidemics in this period have repeated these opportunities many times. And now, in the face of the possibility suggested at the head of this paper, what answer has so-called *scientific medicine* to give as to the nature of the *cause* of cholera? Why, microbes, of course. And is this all it has for answer? If so, and we believe it is, then, we submit, the history of the epidemic of 1884 in Southern Europe and of efforts to check its progress and limit its existence to points first invaded by proceedings based on this assumed nature of the cause proves, by the utter failure to arrest or limit the epidemic by these proceedings, the utter falsehood of the claim set up for these organisms as the producing cause of cholera. They were found in the excreta of cholera patients, and that is just all that is known of their connection with the disease. What of importance attaches to them, if any, in connection with the disease, either as affecting causation or influencing its intensity, or that of any or either of its elements, nothing whatever has been learned from this latest opportunity for their study. But *this* has been learned, that those who have been most engaged in studying these organisms since their proclaimed discovery by Koch, and who have been most earnestly hopeful of a better success in dealing with the plague because of this discovery, have made no better record of dealing with it than has been that of their predecessors before Koch was born. Failure of success here, as elsewhere and heretofore, proves these "scientific" gentlemen to have been spending their industry on barren fields, fields only productive of disappointments, and the great wonder in this connection is that they have not been taught by their failures that they have been looking in a wrong direction for the desired and expected successes they have never realized. Have they been so taught? Not in the least. They still talk of microbes, germs, and disinfectants as glibly and as confidently as if they had once in their lives seen any practical good come to any interest of mankind from all this talk, or proceedings based on it, which, from our present point of observation, revealing only failures, seems to be only so much empty bosh! Why are these men not taught by their own experiences? Is there no lesson in this for us as to the power of pride and conceit to blind the minds even of the intelligent and well meaning? Because they are not instructed, is this any reason why others should not be?

But as to the essential nature of the cause of cholera? We believe we are warranted in saying that, aside from the *effects* of that cause, those who know most of it will readily acknowledge they know very little. But we believe enough is known to prove this cause to be no material thing. It is certain it does not obey the laws of matter.

Then the error which regarded the nature of the cause as material repeated itself by accepting certain suspended secretions and injected mucous surfaces as the disease itself—*i. e.*, consisting in a change of material entities, and before the problem of cure, after these two essential errors, it is not strange that the record of the results of the treatment made by those who have been so misled should come to us as little else than a succession of failures.

The utility of quarantine has been a matter of dispute. Some have held this as useless as a means of arrest of the progress of this fearful scourge. Others claim that its practice has had an efficient and beneficent result, and give what seems to be proof of this. In the present state of our knowledge, there can hardly be a doubt as to the duty of any government where the community is threatened with a visitation of the plague to institute a rigid and careful quarantine. In the absence of any exact knowledge of the essential nature of the cause of that which it is the object of this quarantine to intercept, we may, and we think we ought to, be governed in this matter by what seems proof of the success of the resort in some instances in preserving threatened communities from the ravages of the plague. If this proof be reliable, it is of no material importance to us just what this cause really is, *i. e.*, in deciding this question of quarantine.

But if, notwithstanding the precautions of quarantine, the disease actually appears in a community, then there is present a two-fold duty of doctors and men of the greatest importance. First, to prevent its spread, chiefly pertaining to municipal authority—sanitation—and, second, to cure those who become its victims, and this is expected mainly to be effected by the doctors. In sanitation it is but natural that the authorities to which this more properly belongs should proceed under the advice and direction of doctors. These are supposed to know more as to the factors of the problem of prevention than other men. And here it is that the doctors who claim to be representatives, and exclusive representatives, of "*scientific* medicine" shine, if anywhere, in connection with malignant cholera. But, it is worthy of remark, they shine here as *scavengers*, not at all as doctors. It is not a little curious, nor a little instructive, when we see these men, before the cholera problem, leave, apparently, all thought of the cure of the sick and eagerly expend their energies on *filth!* We say apparently leave thoughts of cure, for the reason that in each succeeding epidemic, instead of having learned in the preceding ones that the means employed by them to this end were not curative at all, and sometimes even worse than useless,* they only continue to repeat these, while the

*In Russia, in 1831, of ninety-three cases treated allopathically, sixty-nine died, or seventy-four and one-fifth per cent. In the same country and epidemic, of forty-nine cases not treated at all, thirty-three died, or sixty-seven and one-fourth per cent, thus showing the probability, at least, that if the ninety-three cases treated allopathically had not been treated at all, seven of them might have recovered. To these seven, certainly, the treatment was worse than useless.

result repeats their failures in the last the same as in the first. It is as though, as to the cure, they had internally confessed judgment of imbecility against themselves, which only made them the more eager to show that there was something in this connection they can do, and hence the rush on filth, to show, if they may, its importance and that they are its masters. If this haste to abandon cure for a warfare on filth surprises us, let it not lead us into any low estimate of the importance of this factor in the cholera problem. And if the seeming imbecility of these doctors as healers be revealed by their abandonment, let us not therefore withhold from them their due meed of praise as the skillful scavengers they often are.

It cannot be denied that filth and squalor appear in some way, perhaps not very well understood, to favor the development of the cholera cause and also the intensity of its action. So that the beneficence of cleanliness is very apparent where this has been made a factor in the combat against this disease. For this reason it is that the advice and labors of these professional scavengers are to be treated as important and with respect. It is only when they cease their work as cleansers and take up the role of explainers that we are compelled to withhold this, and the more and utterly when this brings them to microbes and their belaboring of these as the chief cause of the object of their combat. Where cholera has recently prevailed there has been, under the direction of these professional advisers, much and confident talk of "stamping" these "out," and there has been, consequent on this, very much "stamping" done, but the cholera has been neither less nor less severe therefor. This should be remembered by everybody except doctors. It would seem the lesson is already forgotten by them, for they continue to talk just as confidently of microbes, germs, and disinfectants as matters to confide in as they did before their late signal failures to demonstrate the least benefit from a knowledge of these. They would seem to have gained as little of truth to their theories as of knowledge of better means of cure from their latest opportunity to amend both. They have not yet learned that the fighting of microbes as the cause of cholera is but a "beating of empty straw." Secure cleanliness, and let microbes take care of themselves and amuse the simple, if, indeed, they must still engage themselves with these.

Failing of protection from sanitation, then comes the duty of curing. And how shall this be met? The answer will be determined in great measure by the views of the respondent as to the requirements of the problem of cure, and as to directness or indirectness of the action of the agents these requirements call for. There are two classes of professed healers to whom this question will present itself, and each, as a claim for confidence in his method, may be supposed to appeal to the record his method has made in dealing with past epidemics. Each has a method and record, and each, very likely, will be expecting general approval of his method, perhaps with equal confidence. Let us look at the record of each and see, if we may, which challenges our confidence with greater recorded successes.

One of these parties claims all of advantage which a greater antiquity can give, and this is presented with traditions and maxims with which the centuries have loaded it down, demanding first-class honors for these as well as for the more recent theories by which it has been brought to the use of the indirect means it has employed and in which it still trusts. It knows and trusts only means which it hopes by some indirect action will effect relief for its patients. So it has dealt with cholera through the successive epidemics it has treated, from that of 1831 to that of 1884. Let us look at the record of this indirect method and see how far it merits our confidence. It has employed substantially the same means in all the series of epidemics till this of 1884, when it added to these such as were supposed to be required for the destruction of microbes, now regarded as a new discovery of great importance.

This party of indirect or allopathic practice treated in Russia in 1831 ninety-three cases, of which seventy-four and one-fifth per cent. died. In 1832, of four hundred and fifty-seven thousand five hundred and thirty-six cases, forty-eight and one-half per cent. died. In 1855, in St. Petersburg, of nine hundred and one thousand four hundred and thirteen cases treated, fifty-one and one-half per cent. died. In Russia, in 1831, while seventy-four and one-fifth per cent. died under allopathic treatment, only sixty-seven and one-fourth per cent. died of those who were not treated at all. Of those treated homœopathically, only twenty-one per cent. died. The lowest rate of mortality under the indirect or allopathic treatment was at Canton Rive-de-Gier, 1854, and was thirty-three and one-third per cent. The greatest was as reported from Tischnowitzer Berick, eighty-eight and one-half per cent, while the same report gives the deaths of those under the direct or homœopathic treatment as only five and two-fifths per cent. The reported deaths under allopathic treatment in the epidemics from 1831 to 1884 vary from eighty-eight and one-half per cent. to thirty-three and one-third per cent, the latest, that from

Naples, 1884, being fifty-three per cent., and this with all the aid the doctors could secure from a knowledge of Koch's microbes. It will be seen this is no perceptible improvement over the failures of this method in previous epidemics. It may be added that in reports from four localities a mortality in each of over sixty per cent. is given.

In contrast with these reports, and eloquent in advocacy of the direct method, are the results of its dealings with cholera. In 1854 Dr. Rocco Rubini, in Naples, where the disease has been so signally fatal under the indirect method, treated seven hundred and three cases, three hundred and ninety-one of which were in the Royal Poor-house and in the Third Swiss Regiment, with a loss of but two cases, or two-sevenths of one per cent. This is, so far as I know, the best success on record in treating cholera. In fourteen localities from which we have reports of results of homœopathic treatment of cholera we have the following percentages: Naples, two-sevenths of one per cent.; Munich, in 1836, two and two-ninths per cent.; Canton Rive, 1854, two and a half per cent.; Dr. Adler, two hundred and fifty-five cases, lost four per cent.; Dr. Tripi, in 1854, of six hundred and forty-one cases, lost three and five-sixths per cent. In Austria, in 1832, of twelve hundred and sixty-nine cases, six and two-thirds per cent. died. Dr. Chargé, in Marseilles, 1854, of one hundred and fifty-one cases, four per cent. died. In Champagne, in 1855, of four hundred cases, four and a half per cent. died. In Austria, in 1831, of twelve hundred and sixty-nine cases, six and two-thirds per cent. died. In Genoa, 1854, eight hundred and forty-one cases, eight and a half per cent. died. In 1843, Dr. Rosenberg, of fourteen thousand and twenty-four cases, lost nine per cent. The results of the two methods of treatment, as given above, are mostly from official published reports. These speak for themselves, and speak eloquently, and declare plainly that the indirect, self-styled "scientific medicine" is, in fact, only a therapeutic method *with the "science of therapeutics" left out.*

There is one other relation of the profession of healers to sicknesses, and men liable to be attacked by them, of the greatest importance, greater even than that of healers, by so much as *prevention* is admitted to be better than *cure.* To protect the healthy from attacks of pestilence is certainly better than the most perfect application of the most perfect science by the most perfect skill for its cure;—better, because all suffering of sickness is saved, as is also the chance of failure to cure, always incident to best science and skill, especially in so malignant plagues as is Asiatic cholera, as is seen in the death of two in the seven hundred and three cases treated by Rubini.

How is it, then, with those of this indirect method, who claim to be "scientific," and exclusively "scientific," before this question of prophylaxis? What has their antiquity, their "science," their exclusiveness, their pride, or their conceit to answer to the claims of humanity for protection against the ravages of this scourge? Alas for them! with all their boasting, they have no voice of response to this cry to them for help from the helpless. Before this question of prophylaxis, they are absolutely dumb. It is too true that their long antiquity, with its centuries of opportunities, has taught them nothing. Their boasted science, with the exclusiveness and arrogance of its claims, has taught them nothing. They can say nothing because they have nothing to say. Before this question of prophylaxis their science stands revealed—an empty pretense, and nothing more. Are they shamed by the revelation? They are evidently not at all ashamed. It has taught them neither modesty nor distrust of the method and means by which they have scored so fearful a succession of failures in the epidemics of the present century. Why is it that they have learned nothing of means of protecting humanity exposed to the action of the cause of this so fatal plague? In one word, it is because their boasted science is not science at all. There is in it neither light nor law to point these men to means which can, have, and do protect. They know no law of protection, and therefore are without light or guidance in any search they may make or may have made for the means of prophylaxis, and, therefore, the search, if any has been made, has been without fruit; and so it must ever be till the search is prosecuted in the light and under the guidance of law.

How has it been with the teachers and practitioners of the direct method? Are they equally dumb before this great need of help, which calls to all healers for protection from these so fatal attacks? We have seen the superiority of success which has resulted from the means it has used for cure. Can its practitioners and its means protect from attacks those who are exposed to the action of the cholera cause? No doubt it is within the province of these to protect men as well as to cure them, and herein is one of the brightest jewels in the diadem which history has placed on the head of him who discovered and proclaimed the means both of protection and cure, being guided to the means for both by the light of the universal law which underlies all there is of any "*science* of therapeutics" known to man. Without this law there is no such science, and without this science there is no protection of men from attacks of pestilence. With adequate knowledge of this law and perfect obedience of its requirements, the superiority of this method to the indirect, before the question and duty of prophylaxis, is even greater and more apparent than we have found it to be before the problem of cure.

It was one of the most striking examples of the remarkable insight of Hahnemann which discovered the means of prophylaxis of the cholera pest before he had had opportunity for personal observation of the disease. His absolute confidence in the truth and authority of his discovered law of healing led him, by his logical application of it to the facts of the disease, to say, the most similar remedy will *cure* this so great terror, and the same insight, confidence, and logic enabled him to add, The same similar remedy will also be found to be its prophylaxis. History has abundantly justified both predictions. We have given some of the proofs of this as to the cure. It was our purpose to add statistics of the protection the similar remedy has given from attacks of the disease on the healthy, but we are extending our writing beyond the limits usually permitted to such papers. We will add, however, that in the European epidemic of 1831 the most similar remedies, which Hahnemann declared would both cure and protect, did preserve whole communities, and many of them, when the means were employed as directed, and innumerable individuals to whom the remedies were given were preserved from attacks. Of these last, very few were attacked, and with these the disease was always slight, and of such attacks no one, so far as we are informed, died.

Subsequent epidemics have continued to testify to the sagacity and truth in these prophecies, and to the verity and authority of the law which led to the discovery of this grandest of medical achievements, by continued successes in both cures and protection through the whole series till the present time. We will only add a few instances of the success of the similar remedy in protecting those from attack who were exposed to the cholera poison. The same medicine which in Naples, Italy, cured seven hundred and one of seven hundred and three patients (an unparalleled success, so far as we are informed) was given to near fifty thousand persons. Of these, few were attacked, though they nursed cholera patients or lived in infected houses. Four physicians, viz.: Cigliano, Rubini, Mucci, and Oriati, gave the same medicine to the families of their clientage, numbering about two thousand, and there was no case of cholera or cholerine in them all. The same was true of the Central Home of the

Sisters of Charity and its six hundred inmates, and also of the Typographical Institute of De Angelis; of the workmen and their families, between five and six hundred, not one was attacked.

We have given in this paper but a small portion of the facts which have been given to the public, showing the fearful contrast between the results of the indirect or allopathic method, and the direct or homœopathic method of dealing with this most fatal destroyer of men. The contrast as to the ratio of cures to cases treated is not a little startling, the one *curing* seven hundred and one in seven hundred and three cases, the other *losing* eighty-eight and one-half of every hundred treated. These are the extreme figures of the reports here given. They are, beyond cavil, reports of experienced facts. Is there no duty as to reception of these facts and the instruction they bring us? Have they been known, these facts of the direct method, to the gentlemen of the indirect, who have lost, in the epidemic of 1831 and subsequent ones, more than fifty per cent. of all they have treated? No doubt they have been known. Then why have not these gentlemen been instructed into this truth of the better method, which there can be no doubt would have given to them, as it has to others, a better record? Because they *hated* this truth, and would only shut their minds against its reception. They had hated, opposed, and maligned it before the cholera came to testify to its truth by submission to its master, and now, before the record of its success and their own failures, shall they humble themselves to accept from this hated and maligned truth a knowledge of a way better than their own? How can they escape from this testimony, which stares them in the face from the records of every epidemic—their own and that of this hated truth? How can they bear the contrast history holds up to the world, of success on the one side and failure on the other, and the success not their own? They have one escape, and only one, which has ever been, and is, the resort of the weak and the wicked, when they have no other, in these two words: *non credo.* But is this an escape or only a delusion which so often follows hatred of the truth? Is not this rejection of the truth a proof of the unity of human character and nature in all time? Is it not true now of these as it was of old of those of whom it was said, "*Because* I tell you the truth, ye believe not?" Is it not the same hatred of truth in this province of it which, in another, crucified the Son of God to be rid of His presence on earth, which now would destroy it and its record by consigning both to the limbo which swallows up rejected truths here and now, and the rejectors of them hereafter?

But do these doctors really know the facts of the record? No doubt they do. The fact of the cure of seven hundred and one of the seven hundred and three cases, with a statement of the means by which this unparalleled success was attained, was sent in a circular to every doctor in the city of Naples. Did they regard this? Not at all! They went on, as others had before, and made a record of fifty-three per cent. of deaths of all treated. Are these men other than criminals before God and man, and when called to answer, as men will be, for what they have done and for what they have neglected to do, will their *non credo* be accepted as a plea of justification?

But there is other, and if possible stronger, evidence of a more aggravated wickedness in the expulsion from the pharmacy of one of the large cholera hospitals, in the late epidemic of 1884, of the very medicine which had cured those seven hundred and one sick. They would make it impossible that any patient there should have the hated drug—hated only because it cured, and because the hated Hahnemann had first pointed to it as the specific for cholera. While expelling this most efficient and beneficent remedy from their premises, were they not, remembering the origin of the proclaimed specific relation of the drug to the disease and him who proclaimed it, actuated by the same spirit as were those of old who spurned the healed of blindness with the proud and arrogant query—"*Dost thou teach us?*"

P. P. WELLS.

